# Enhancing Surveillance Systems: Integration of Object, Behavior, and Space Information in Captions for Advanced Risk Assessment

**DOI:** 10.3390/s24010292

**Published:** 2024-01-03

**Authors:** Minseong Jeon, Jaepil Ko, Kyungjoo Cheoi

**Affiliations:** 1 Department of Computer Science, Chungbuk National University, 1 Chungdae-ro, Seowon-gu, Cheongju, Chungbuk 28644, Republic of Korea; jeonminseong@chungbuk.ac.kr; 2 Department of Computer Engineering, Kumoh National Institute of Technology, 61 Daehak-ro, Gumi, Gyeongbuk 39177, Republic of Korea; nonezero@kumoh.ac.kr

**Keywords:** surveillance system, image captioning, descriptive captions, risk assessment, BLIP-2, BERT

## Abstract

This paper presents a novel approach to risk assessment by incorporating image captioning as a fundamental component to enhance the effectiveness of surveillance systems. The proposed surveillance system utilizes image captioning to generate descriptive captions that portray the relationship between objects, actions, and space elements within the observed scene. Subsequently, it evaluates the risk level based on the content of these captions. After defining the risk levels to be detected in the surveillance system, we constructed a dataset consisting of [Image-Caption-Danger Score]. Our dataset offers caption data presented in a unique sentence format, departing from conventional caption styles. This unique format enables a comprehensive interpretation of surveillance scenes by considering various elements, such as objects, actions, and spatial context. We fine-tuned the BLIP-2 model using our dataset to generate captions, and captions were then interpreted with BERT to evaluate the risk level of each scene, categorizing them into stages ranging from 1 to 7. Multiple experiments provided empirical support for the effectiveness of the proposed system, demonstrating significant accuracy rates of 92.3%, 89.8%, and 94.3% for three distinct risk levels: safety, hazard, and danger, respectively.

## 1. Introduction

In various sectors of contemporary society, a plethora of closed-circuit television (CCTV) cameras have been strategically deployed to monitor and record various safety incidents and criminal activities. The growing concern for societal safety has led to an increasing demand for CCTV and security surveillance. According to The Business Research Company’s “Global Security Surveillance System Market Report 2023” [1], the global security surveillance system market is forecasted to expand from USD 130.08 billion in 2022 to USD 148.29 billion in 2023, at an annual growth rate of 14.0%. With the escalating need for safety in high-risk areas, it is anticipated that the surveillance system market will reach a substantial USD 230.47 billion by the year 2027.

Surveillance systems are engineered to identify patterns within monitored scenes, encompassing risky behavior, abnormal actions, and incidents. Extensive research has been conducted on these systems, primarily focusing on the detection of abnormal behavior such as ‘fighting’ and ‘fainting’. This involves the utilization of technologies such as object detection and recognition, tracking, pose estimation, movement detection, and anomaly detection of objects [2,3,4,5,6,7,8,9]. Jha et al. [8] proposed an N-YOLO model designed for the detection of abnormal behaviors, such as fighting. This model tracks the interrelationship of detection results in subimages, integrating them with the inference outcomes through a modified YOLO [10]. In a related study, Kim et al. [9] introduced the AT-Net model, specifically designed for abnormal situation detection. The model aims to mitigate classification ambiguities and minimize information loss by integrating object detection and human skeletal information.

These various studies [2,3,4,5,6,7,8,9] have improved performance by incorporating diverse feature information related to abnormal behavior. Nevertheless, conventional methods exhibit certain limitations. Firstly, these methods do not comprehensively consider the spatial context associated with abnormal situations. It is crucial to recognize that, even with identical objects and behavior, interpretations may vary significantly based on the spatial context, resulting in different responses to specific risk scenarios. For instance, the action of sitting on a bench, compared to sitting on a railing or cliff—both categorized as sitting actions—results in distinct risk levels. Sitting on a bench is considered safe, whereas sitting on a cliff represents an extremely dangerous situation. Secondly, within the framework of current surveillance systems designed to detect potentially hazardous scenarios, the outcomes are typically displayed as alerts on the monitoring screen or delivered as recorded footage featuring the identified risk elements. However, in numerous instances, actions exhibiting a higher degree of movement compared to the surrounding behavior might attract attention but may not necessarily be indicative of an actual accident. For instance, conventional surveillance systems may flag regular running in a park as abnormal since it involves more movement than walking or sitting. Consequently, further scrutiny becomes imperative for the observer to re-evaluate the scene and interpret the situation accurately in order to discern the precise nature of the detected abnormal behavior. Ultimately, for a more accurate and prompt interpretation of risk situations, surveillance systems must not only consider the actions of the detected objects but also take into account the surrounding environment and situation, assess the overall risk level, and provide the observer with specifically interpreted information. Thirdly, surveillance systems should have the capability to detect and interpret a broad range of risk elements and accidents, without being limited to specific locations or behaviors. For example, a system designed to detect instances of drowning in specific locations, such as swimming pools, may efficiently identify dangerous situations by employing technologies like object detection and movement-based anomaly detection, incorporating learned behavior patterns associated with drowning. However, in the case of CCTV systems installed in public spaces or on the streets, which necessitate versatile detection capabilities, the spatial context required for detection differs from that in the pool example mentioned above. Numerous categories of risks manifest in a variety of environments, involving hazardous elements such as fires and safety-related incidents like traffic accidents or altercations. Relying solely on specific technologies presents a considerable challenge in detecting a wide array of situations and types, consequently limiting the applicability of conventional methods in surveillance systems. In summary, surveillance systems must have the capability to comprehensively leverage spatial information, convey data in a format that can be rapidly and accurately grasped by the observer, and represent learnable data for potential risk factors in diverse situations. To address this challenge, this paper introduces a novel approach for a surveillance system.

Humans utilize a structured and high-level expressive tool, namely, language. Applying human-friendly natural language to the surveillance system can effectively address the aforementioned challenges. A situation to be detected by the surveillance system can be articulated in sentence form using natural language. Natural language can be employed to express a scene’s danger, explain an accident in sentence form, and elucidate an accident in sentence form, encompassing various information such as the characteristics of the object, the presence or absence of a person, the distinction between adults and children, the type of accident, the place of occurrence, and nearby risk factors. When the scene is conveyed through such sentences, the observer can promptly comprehend the situation.

The rapid advancement of natural language processing (NLP) technology has paved the way for the utilization of words and sentences as data for learning, leading to the development of various models. Large language models (LLMs), in particular, which have been trained on extensive language datasets, exhibit remarkable performance in a multitude of natural language processing tasks. These large language models are proficient in diverse tasks such as sentence generation, translation, text summarization, and question answering.

Image captioning is a subfield of NLP that involves generating text describing the content of an image in natural language. By incorporating image captioning technology, enhanced with large language models boasting high generalization performance, into surveillance systems, it is possible to represent images through information-rich sentences. Notably, image captioning technology proves to be exceptionally well suited for the detection of hazardous behavior and the analysis of accident scenarios. This is attributed to its capacity to offer a detailed representation of the scene, encompassing information about individual characteristics, actions, and the spatial context. Furthermore, by enabling the system to autonomously assess risk levels for captions (sentences) generated through image captioning technology, proactive mitigation of safety incidents becomes feasible, allowing for prompt and accurate responses to any accidents that may occur.

In this paper, we introduce a novel surveillance system designed to surpass the limitations of traditional surveillance systems, which are frequently restricted to a primary emphasis on object-centric behavior analysis. Our system generates descriptive captions that encompass details regarding objects, actions, and spatial context extracted from surveillance target footage. These captions are subsequently utilized to assess the risk level of the observed scene. To generate captions for surveillance scenes, it is necessary to construct a dataset comprised of [Image-Caption-Danger Score]. The dataset should feature caption data presented in a novel sentence format, deviating from conventional caption structures, and should encompass a myriad of information encompassing objects, actions, and spatial context, facilitating comprehensive interpretation. To facilitate the interpretation of scenes by the image captioning model, we utilized BLIP-2 [11], a large language model well regarded for its efficiency in handling multimodal tasks while minimizing parameters and training costs, all while delivering state-of-the-art performance. We fine-tuned the BLIP-2 [11] model with the newly-constructed dataset to guide it in generating captions that adhere to the newly-defined sentence structure. Subsequently, we utilized BERT (bidirectional encoder representations from transformers) [12] to interpret the semantic content of the generated sentences and assess the risk level associated with each scene.

The overall structure of this paper is as follows. In Section 2, we delve into general research related to image captioning, existing studies on surveillance systems that incorporate image captioning, and the exploration of LLMs. Section 3 provides a detailed description of our newly constructed dataset and the overarching system structure. Section 4 presents a comprehensive evaluation of the proposed system’s performance, utilizing both quantitative and qualitative analyses. Additionally, we delve into potential avenues for system enhancement through result analysis. Finally, in Section 5, we conclude the paper by summarizing the proposed system, discussing future development directions, and outlining potential areas for further research.

## 2. Related Work

Image captioning is a technology that generates descriptive captions for an input image. Notable studies in this domain are referenced as [13,14,15,16]. Vinyals, O. et al. [13] proposed a method that connects an encoder constructed with convolutional neural networks (CNN) for extracting image information with a long short-term memory (LSTM) [17] decoder for caption generation. Xu, K. et al. [14] introduced an approach that enhances the relationship between images and captions by incorporating an attention mechanism. Liu, W. et al. [15] suggested an image captioning technique that sequences images to serve as inputs for the transformer [18] model. Wang, P. et al. [16] proposed an integrated system that utilizes multimodal pre-training to represent data in a unified space. This approach allows for the expression of both image and language information in patches, facilitating simultaneous processing of image and language information within the transformer.

In the domain of surveillance systems incorporating image captioning, Dilawari et al. [19] introduced a system utilizing the VGG-16 [20] to extract specific situational information from videos and generate captions through a bidirectional-LSTM [21], with a primary focus on object-related attributes. W. Chen et al. [22] proposed a system that computes anomaly scores by combining captions generated using SwinBERT [23] and video features extracted via the ResNet-50 architecture [24]. These studies often rely on datasets like UCF Crime [25], NTU CCTV-Fights [26], ShanghaiTech [27], and XD-Violence [28], encompassing diverse types of behavior, including fighting, fainting, loitering, and abandonment. However, a noteworthy limitation arises from the prevalent lack of comprehensive captions within these datasets, which typically offer basic object descriptions but fail to capture nuanced object behavior or contextual space information. Consequently, despite the application of image captioning to enhance surveillance systems, the resulting captions primarily center on objects, neglecting the vital space context crucial for a comprehensive scene risk assessment. This limitation underscores the need for further research in integrating space information to enhance the efficacy of surveillance systems.

The advent of LLMs has brought about substantial advancements in NLP, enhancing the capacity to understand and generate human language by discerning word similarities and contextual relationships, and effectively handling sentence structure, grammar, and meaning. 

Prominent models in the domain of LLMs include BERT [12], GPT [29], T5 [30], and LaMDA [31]. BERT [12] is a deep learning-based model in NLP distinguished by its capability to extract bidirectional contextual information from extensive volumes of raw text, capturing sequential relationships between sentences and representing words and their context in vectors. This comprehensive approach empowers BERT to consider both preceding and subsequent text, granting it an extensive understanding of language. Originally designed for language comprehension, BERT has significantly advanced the field of NLP. Furthermore, the LLM landscape boasts a multitude of models, such as GPT [29] and T5 [30], which have demonstrated remarkable performance in tasks such as sentence translation and summarization. Additionally, LaMDA [31], developed for interactive applications, represents another notable addition to the suite of LLMs. These models collectively signify the diverse and expanding capabilities of LLMs in the field of NLP.

In the domain of LLMs, a notable constraint is their inherent inability to comprehend image features due to the absence of image data during their training process. To overcome this limitation, extensive research is being conducted on large multimodal models (LMMs) that enrich LLMs with image information to establish a connection between images and text. These LMMs are pre-trained on a massive scale of diverse data types, including text, images, audio, and video, thereby equipping them with the capacity to perform a multitude of tasks, ranging from image captioning to vision question answering (VQA). Prominent systems, such as BLIP-2 [11], OpenAI’s GPT-4 [32], Google’s Gemini [33], and LAVA 1.5 [34], are all founded upon these large multimodal models, demonstrating the growing significance of this approach. Furthermore, a prevailing trend in the field involves the utilization of large web datasets for multimodal training, resulting in the development of a multitude of models. For instance, BLIP-2 [11] is trained on an extensive dataset comprising image–text pairs gathered from the web. This model incorporates frozen pre-trained models in both its encoder and decoder and effectively addresses the modality gap between the encoder and LLMs through the query transformer (Q-Former), achieving remarkable state-of–the-art (SOTA) performance in various vision–language tasks while demonstrating significant zero-shot capabilities.

In this paper, we present a surveillance system tailored to address the inherent constraints of prevailing surveillance systems. Our approach involves the creation of a novel dataset specifically tailored for surveillance applications, encompassing comprehensive captions that incorporate object information, action details, and space context. To further enhance the interpretative capacity of this surveillance system, we fine-tune BLIP-2 [11], a model trained on extensive web data, utilizing our newly curated dataset. Additionally, we leverage BERT [12] to comprehend the semantic nuances of the generated captions, enabling us to quantify the risk level based on this refined interpretation. This combination of novel dataset construction, fine-tuning, and semantic understanding represents a significant step toward a more effective and context-aware surveillance system.

## 3. Methodology

**Figure 1:**
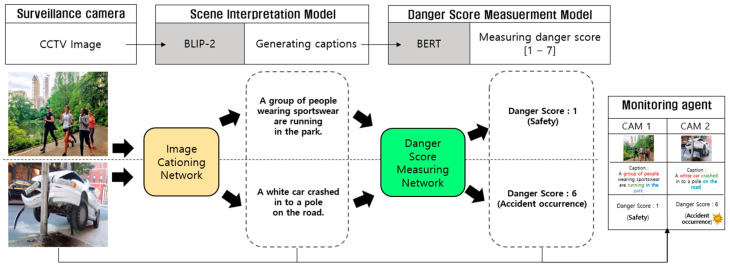
The comprehensive workflow of the proposed system illustrates the entire process flow, encompassing scene interpretation and danger score measurement, while showcasing the results generated at each stage.

Figure 1 depicts a schematic representation of the proposed system’s comprehensive workflow. The system initially takes video input from CCTV sources. Subsequently, this video data undergo processing through an image captioning network, generating interpretative captions to elucidate the content of the scenes. These captions encompass a wealth of details, encompassing object attributes, behaviors, and spatial context. Following caption generation, a specialized risk assessment module conducts an in-depth analysis of these generated captions. This module analyzes the information embedded within the captions, enabling a thorough evaluation of the scene’s risk level. 

In Figure 1, a pair of surveillance images depicting different levels of risk is presented as the input: the first depicts people running in the park, and the second depicts a car accident scenario. The deployed image captioning network effectively formulates precise captions corresponding to the content of each video, which are subsequently evaluated by the risk assessment module to determine the pertinent risk levels. In this evaluation, the first image, portraying a safe situation, is assigned a danger score of ‘1’, while the second image, depicting an actual accident, is associated with a danger score of ‘6’. The danger scores defined within our proposed system are categorized into seven distinct stages, with ‘1’ indicating a safe situation, and higher scores indicating escalating levels of risk. For an in-depth elucidation of the danger score methodology, please refer to Section 3.1. This systematic approach enables the precise evaluation of risk levels in different surveillance scenarios.

### 3.1. The Construction of the Dataset

The proposed system utilizes the large multimodal model BLIP-2 [11]. While BLIP-2 [11] has undergone pre-training on a diverse dataset encompassing various natural language processing tasks, its inherent design lacks task-specific optimization. Therefore, to achieve appropriate results when applying the large multimodal model to the proposed surveillance system, fine-tuning is imperative. 

Conventional training datasets for surveillance systems often lack interpretive captions, and when available, the captions typically offer only rudimentary object descriptions, lacking comprehensive information about object actions or the surrounding space context, essential for practical surveillance applications. Therefore, we have constructed a dataset incorporating detailed information regarding objects, behavior, and spatial context. Additionally, our dataset encompasses diverse environmental conditions, including overcast days, nighttime shooting, and low-light environments, ensuring robustness in the face of changes due to various shooting conditions. Furthermore, a dataset is specifically created to detect and interpret individuals even when they are at a distance from other objects, focusing on people who are the subjects of the monitored safety incidents. This ensures the delivery of sufficient information for an accurate understanding and analysis of the monitored situation, surpassing the mere listing of visual elements. Additionally, this approach significantly aids in identifying and analyzing essential elements, individuals, for risk assessment within the visual field. As a result, a total of 2741 comprehensive datasets were built to enable BLIP-2 [11] to generate relevant captions for the surveillance system. Additionally, these datasets facilitate risk assessment following the processing of captions through BERT [12]. The danger score here represents a numerical value corresponding to the risk level, with specific classification criteria provided in Table 1, thereby facilitating the quantification of risk levels in the surveillance context. The construction of this comprehensive dataset is instrumental in enhancing the suitability and effectiveness of the proposed system for real-world surveillance applications.

### 3.2. Definition of Risk Level and Danger Score

The classification of risk levels for input images involves three fundamental categories: safe situations, hazardous situations, and accident occurrences, each being assigned distinct danger scores. Table 1 illustrates that the risk levels representing safety and hazards are divided into two stages each, whereas the danger level is further segmented into three stages, contingent upon the varying levels of risk. This methodical classification system serves to delineate the entire range of risk across diverse surveillance scenarios.

The ‘Safety’ risk level typically involves routine activities such as walking, sitting, or running in secure areas like pathways, parks, or indoors. However, it is essential to note that even within the same risk level, situations involving children may inherently pose a relatively higher level of risk compared to those involving adults. Consequently, when adults are engaged in these activities, a danger score of ‘1’ is assigned, while the presence of children is associated with a higher danger score of ‘2’, signifying the increased potential for elevated risk in such scenarios. This nuanced approach enables a more accurate evaluation of potential danger in the presence of children during actions categorized as ‘safe’.

The ‘Hazard’ risk level encompasses routine activities that typically occur in safety situations but are contingent upon the context of the surrounding space. For instance, the act of sitting is considered safe when performed on a bench, meriting a danger score of ‘1’; however, if the same action takes place on a railing, bridge, or roof, which inherently pose higher risks, it is reclassified as hazardous and is assigned a danger score of ‘3’. This systematic approach ensures that the level of risk is appropriately assessed in various space contexts, thereby enhancing the precision of the surveillance system.

The ‘Danger’ risk level is designated for detected actions categorized as accidents, encompassing scenarios where individuals have collapsed or incidents involving activities such as fights, fires, or traffic accidents. The degree of danger, as quantified by the danger score, escalates when these actions occur in hazardous environments like railings, cliffs, roads, or construction sites. Furthermore, a higher danger score is allocated when such actions involve children. This systematic risk assessment approach enables a more nuanced and accurate evaluation of the risk level within situations classified as dangerous, accounting for the influence of environmental factors and the age of the individuals involved.

Table 2 provides a comprehensive overview of the dataset distribution, systematically organized according to distinct risk levels. A total of 2741 images were collected, each necessitating a thorough analysis of object attributes, behavior, and spatial context. These images were meticulously selected in accordance with the classification criteria outlined in Table 1. The captions accompanying these collected images were meticulously crafted to delineate the distinctive characteristics of the object type, elucidate the actions of the object, and describe the space context in which the object is situated. Subsequently, datasets were systematically constructed by assigning an appropriate danger score to each situational scenario, ensuring the comprehensive representation of various risk levels in the surveillance system.

### 3.3. The Sentence Structure Format of Captions

During the dataset construction phase, careful consideration was given to the design of caption structures. These structures were intentionally designed to include essential components, specifically, the object type, object attributes, object behavior, and the spatial context. To offer further clarity and insight into the employed caption structure within the dataset, Table 3 presents illustrative examples.

The captions presented in Table 3 meticulously delineate the specifics of objects within surveillance footage. Objects are discerned by red highlights, with each highlighted section indicating the object’s category and pertinent details. This information encompasses the identification of whether the subject is a person, a vehicle, or a fire, and further specifies whether the person is an adult or a child, along with details about their attire. Green highlights are employed to depict the behavior of the object, while blue highlights convey information pertaining to the spatial context. For instance, the first and second examples within Table 3 both depict an adult male engaged in walking. However, the contextual differences between these examples are significant. The first example takes place in a park, characterized as a safe environment. In contrast, the second example involves walking on a road, an action considered perilous due to jaywalking. Additionally, the third and fourth examples depict a fainting incident, classified as an emergent situation within the danger (accident occurrence) category. The third example takes place within a room, devoid of additional environmental hazards, resulting in an assigned danger score of ‘5’. In contrast, the fourth example occurs on a road frequented by cars, posing a higher risk due to the increased likelihood of subsequent accidents and is thus assigned a danger score of ‘6’. This meticulous approach to caption structuring ensures the effective capture and conveyance of comprehensive information regarding the objects, their actions, and the relevant spatial context. Consequently, it enhances the dataset’s applicability in the surveillance system.

### 3.4. Scene Descriptive Caption Generation and Risk Assessment

**Figure 2:**
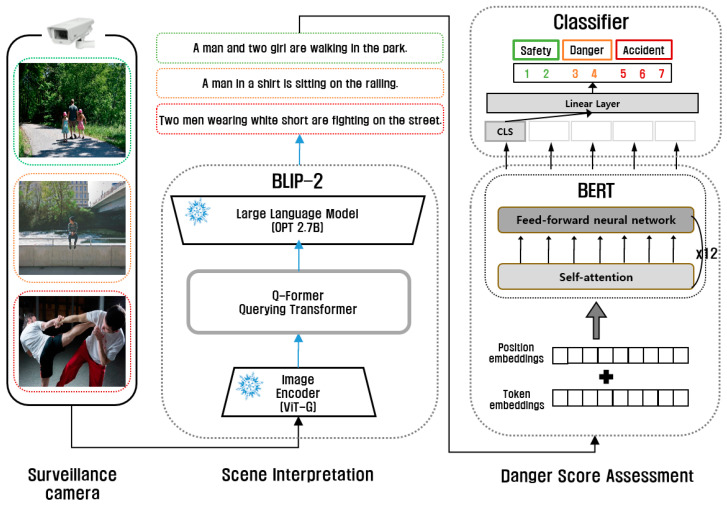
The architectural framework of our proposed system: the figure illustrates the procedural sequence wherein CCTV video input undergoes processing via the BLIP-2 model to produce captions, and these captions are subjected to analysis by the BERT model to facilitate risk classification.

The architectural framework of the proposed system is illustrated in Figure 2. The system follows a two-step process: Firstly, it fine-tunes BLIP-2 [11] using the dataset specifically tailored to generate descriptive captions that interpret scenes. BLIP-2, an advanced model in image captioning, combines visual and textual data, making it adept at understanding and describing complex scenes in surveillance footage. This model’s strength lies in its ability to contextualize visual elements within the framework of natural language, offering a more nuanced interpretation than traditional image recognition models. Subsequently, BERT [12] is employed to perform a semantic analysis of these captions. BERT’s key feature is its bidirectional training, allowing it to understand the context of a word based on all of its surroundings in a sentence. This is a significant departure from previous models that processed text in one direction, either left-to-right or right-to-left, which could overlook the broader context of certain words or phrases. BERT’s deep understanding of language nuances makes it particularly effective in assessing the risk levels in the captions generated by BLIP-2. Following the analysis, the system conducts a comprehensive assessment of the risk level associated with each scene. The risk levels are categorized and quantified on a scale ranging from 1 to 7, reflecting the severity of the risk. This multistage approach is a crucial component of the system’s capacity to deliver a nuanced evaluation of scene risk levels, significantly enhancing the efficacy of the surveillance system.

In the second image of Figure 2, the BLIP-2 framework is depicted, featuring three fundamental components: the image encoder, the query transformer (Q-Former), and the large language model (LLM). The query transformer is a trainable module designed to bridge the gap between the fixed-weight image encoder and the large language model. It utilizes ViT-G [35] for the image encoder and OPT 2.7B [36] for the large language model. BLIP-2 [11] undergoes fine-tuning using the constructed dataset, a process known to be computationally intensive. To mitigate the associated costs, the LoRA (low-rank adaptation) [37] technique, as proposed by Hu, is implemented in this system.

The analysis of the semantic content within the generated captions is facilitated by the utilization of BERT [12]. As illustrated on the right side of Figure 2, the scene’s risk level is subsequently measured through a classifier. BERT represents a significant milestone in the domain of natural language processing, distinguished by its exemplary performance across a spectrum of language-related tasks. Diverging from its predecessors, BERT employs a bidirectional language model that comprehensively evaluates each word within a sentence, resulting in a more profound understanding of context. The special CLS token, situated in the upper right portion of Figure 2, consistently appears at the start of BERT sequences. This token serves to encapsulate the overarching context of the input sequence and is particularly well suited for classification tasks. The proposed system is crafted to have captions generated by BLIP-2 to produce a CLS token through BERT. The vector derived from this CLS token then undergoes further processing via a linear layer, culminating in classification that determines the scene’s risk level and associated danger score using a softmax function. The combination of BLIP-2 and BERT in the proposed system leverages the strengths of advanced image processing and deep language understanding. This synergy results in a sophisticated risk assessment tool capable of interpreting complex surveillance scenarios with a high degree of accuracy.

## 4. Experiments and Results

### 4.1. Experimental Setup

The experimental setup for evaluating the proposed system is outlined as follows. BLIP-2 [11] is configured with an input image size of 224 × 224, utilizing a fixed batch size of 16. The optimization process is executed using the Adam [38] optimizer, and fine-tuning takes place over a span of 50 epochs. The learning rate is set at 10^−5^. To facilitate the generation of content-rich sentences, the model’s maximum output length is set to 30 characters. For BERT [12], the learning rate is established at 10^−5^, and a batch size of 32 is employed. BERT undergoes fine-tuning over 100 epochs. The experimental dataset comprises a total of 2741 images, distributed into training, validation, and test subsets at an 8:1:1 ratio. All experiments are conducted on a single A100 GPU.

### 4.2. Experimental Results and Analysis

#### 4.2.1. BLEU Score for Generated Captions

BLIP-2 [11] underwent fine-tuning to generate sentences containing object attributes, actions, and space context, as exemplified in Table 3. To assess the system’s capability to produce captions adhering to the prescribed structural format, encompassing object characteristics, actions, and space information, we computed the BLEU (bilingual evaluation understudy) score [39]. The BLEU score is the most widely used metric in the field of machine translation evaluation, serving as a quantitative index to measure translation quality. It gauges the degree to which the terms within machine-generated candidate sentences align with those in reference sentences, with penalties applied to lengthier candidate sentences. The resultant BLEU score is presented as a numerical value within the range of 0 to 1, with higher scores signifying improved sentence generation quality. This multifaceted BLEU scoring approach ensures a nuanced evaluation of sentence generation performance.

The evaluation of caption quality generated by BLIP-2 was carried out using the BLEU score, with the frequency of generated captions as the underlying criterion for evaluation. The results of the BLEU scores for our dataset are outlined in Table 4. Notably, our newly constructed dataset, despite incorporating less common terminology related to risk scenarios, accidents, and space contexts, boasts an impressive BLEU-4 score of 0.4096. This accomplishment serves as a testament to the effectiveness of our dataset’s captions in fulfilling their designated role. Furthermore, it reaffirms the successful fine-tuning of BLIP-2 using our dataset, ensuring the generation of pertinent captions suitable for the surveillance system.

#### 4.2.2. Qualitative Evaluation of Generated Captions

Table 5 presents a comprehensive comparative analysis of caption generated results between the proposed system and existing image captioning models. The comparative models encompass state-of-the-art models developed following the emergence of transformer-based approaches, including CPTR by Xu, K [15], OFA by Liu, W [16], and BLIP-2 by Li, J. [11] (before fine-tuning). Each of these models [11,15,16] has undergone pre-training on distinct datasets. Specifically, CPTR [15] in Table 5a was pre-trained utilizing the COCO dataset [40]. OFA [16] in Table 5b was pre-trained using a diverse range of datasets, including CC12M [41], CC3M [41], SBU [42], COCO [40], and VG-Cap [43]. Additionally, BLIP-2 [11] in Table 5c was pre-trained on the LAION dataset [44]. Table 5d signifies the BLIP-2 [11] model fine-tuned using the self-constructed dataset proposed within this paper.

Table 5a illustrates a scene featuring a pedestrian jaywalking on a roadway, including the potential accident hazard. Upon examining the generated captions, it is evident that CPTR and BLIP-2(base) produce an incorrect caption, failing to accurately detect the object. Meanwhile, OFA produces a caption lacking essential human-centric information. The results obtained from our model, showcase success in generating a caption that comprehensively incorporates essential details regarding the individual, the walking action, and the spatial context of the road.

Table 5b,c illustrates a scene featuring a person who has fallen due to an accident. Upon examining the results for the image in Table 5b, all models produced captions that showed a person lying down. However, it is noteworthy that only our model was able to produce captions offering detailed information about the object and its surrounding environment. Other models misrepresented space information or failed to adequately represent the object. CPTR and OFA generate interpretations centered around a person standing nearby, deviating from a primary focus on the fallen individual. BLIP-2(base) focuses on the fallen person but is observed to lack information related to space and objects. In contrast, presenting results from our model, it accurately formulates a sentence that encompasses contextual spatial details, including the fallen person, car, and road. Notably, it surpasses anticipated outcomes by also providing a descriptive account of the person standing nearby. 

Table 5d illustrates a scene featuring two men engaged in a physical altercation. However, CPTR and OFA inaccurately capture the action-related information. BLIP-2(base) generates the correct caption, yet it is perceived as lacking in expressiveness. In contrast, presenting results from our model, generates a caption that accurately describes both the specific object information of the two men and the actions “fighting” and “in the room”. These experiments have demonstrated that a model fine-tuned with a dataset specialized for surveillance systems can indeed generate captions effectively, aiding in the interpretation and judgment of scenes. Furthermore, the results underscore that existing benchmark datasets may not be well suited for specific tasks, highlighting the importance of developing task-specific datasets.

#### 4.2.3. Risk Assessment Results

Table 6 presents the results of risk assessments conducted by our system. Specifically, Table 6a,b pertain to safe situations, exemplified by individuals walking on a path. Our proposed system has assessed both scenarios as ‘safe,’ but it distinguished between them by assigning a danger score of ‘1’ to Table 6a and ‘2’ to Table 6b. It is noteworthy that Table 6b receives a danger score of ‘2’ primarily due to the presence of a child in the scene. The inclusion of a child is considered to increase the assessed danger score compared to Table 6a, where no child is present. 

Table 6c,d showcase images portraying individuals engaged in potentially hazardous behavior, specifically sitting on railings. While the action of sitting can be detected in situations classified as safe, the change in space context contributes to the classification of danger level risk. Table 6c generates a successful caption, offering a descriptive account of two women seated on a railing. As a result, the system accurately assessed the danger score as ‘3’. Similarly, Table 6d generates a precise caption, featuring the object ‘girl’ and spatial context of ‘railing’. Given that a child, rather than an adult, is detected in the scene, the system assigned a danger score of ‘4’. This exemplifies the system’s proficiency in distinguishing between actions that may appear similar but carry varying levels of risk, underlining its capacity to conduct precise risk assessments in diverse situations. 

Table 6e,f,g feature images with people who have collapsed. The action of collapsing is inherently associated with the classification of danger (=accident occurrence), which, in turn, triggers a danger score of ‘5’ or higher. The precise danger score is contingent upon several factors, notably the space context and whether the affected individual is an adult or a child. Table 6e portrays a woman collapsing on the indoor floor. Since the indoor floor space does not add additional accident risk, the system assigned a danger score of ‘5’. Conversely, Table 6f also depicts a collapsing action similar to that in Table 6e, but it takes place on outdoor stairs rather than indoors. The system intelligently infers that the ‘stair’ area poses a higher level of risk compared to the ‘indoor floor,’ assigning a danger score of ‘6’. In Table 6g, a child is observed collapsing outdoors. The subject is identified as a child, and the incident occurred in an outdoor setting where there may be additional risk. This crucial distinction prompts the system to assign a danger score of ‘7’, emphasizing our system’s capacity to recognize differences in risk based on the age of the individual involved and the specific spatial context.

As shown in Table 7, the proposed system exhibited remarkable accuracies of 92.9%, 89.5%, and 94.3% for the three distinct risk levels: safety, hazard, and danger (=accident occurrence), respectively, during the evaluation involving 301 test data samples. These results affirm the valuable nature of the proposed dataset, providing easily accessible information essential for the precise evaluation of risk levels.

Moreover, the consistent achievement of high accuracies across all risk categories underscores the robustness of the system. Additionally, the model trained with the proposed dataset demonstrates the system’s ability to adequately interpret the relationships between types of objects and behaviors in various locations, further confirming its effectiveness and reliability in real-world applications.

To integrate the proposed scene interpretation and danger score measurement system into actual surveillance systems, it is necessary to conduct experiments to verify its proper functioning under adverse conditions, including various lighting scenarios, noise, and weather. Table 8 presents experimental results demonstrating the robustness of the proposed surveillance system in challenging shooting environments influenced by factors such as lighting, shadows, and weather. Table 8a–c utilizes an image of the same accident pattern captured under different environmental factors, including low resolution, nighttime darkness, and obscured objects due to shadows. In Table 8a, a man has collapsed on the road, captured using a low-resolution camera. Despite the small size of the individual and the low resolution of the image, the proposed system accurately interpreted the fallen man and predicted the correct level of risk. Table 8b depicts a nighttime traffic accident scene where a man is hit by a car in dark conditions. The system successfully detected and interpreted the incident involving the man and the car. Table 8c includes a scene where a man has collapsed under a tree’s shadow, obscuring the view. Even in this scenario, the system effectively detected both the car and the fallen man, correctly interpreting the accident scene centered around the fallen individual. These results highlight the system’s capability to operate robustly using datasets collected from various environments and emphasize its applicability as a reliable surveillance tool, even in changing shooting environments and amidst obstructive factors.

Table 9 presents instances of distinct types of limitations and areas for improvement encountered by our proposed system. The cases in Table 9 are examples classified under the “Danger” category.

Table 9a,b were misclassified as hazard and safety, respectively. In Table 9a, the scene depicts a man who has fallen off his bicycle. The generated caption by our system primarily emphasized a safety officer standing in proximity to the fallen individual rather than the person in distress. Given the safety officer’s placement on the road, the system classified the area as hazardous, assigning a danger score of ‘3’. The system’s misinterpretation in this instance can be attributed to the camera angle, which caused the primary subject (the fallen adult male) to appear relatively smaller compared to other objects in the vicinity, notably the standing safety officer. Table 9b portrays a scene featuring a boy who has collapsed due to an accident. The system interpreted this scene as a boy sitting on the ground and accordingly assessed the danger score as ‘2’. It is essential to emphasize that the actual scenario involves a boy who has fallen subsequent to a bicycle accident. This example underscores the inherent limitations of image-based systems, as the concept of action depends on continuous information, which remains beyond the reach of static images. The errors discerned in Table 9a,b, where the object size was either inadequately small or entailed unrecognized words, underline the potential for system improvement through the accrual of more diverse data, encompassing a broad spectrum of object sizes and textual information. Training the system with this augmented dataset holds promise for addressing issues related to caption generation across a multitude of scenarios. Nevertheless, it is imperative to acknowledge the intrinsic limitation associated with the detection of intricate behavioral patterns at the single-frame level. A solution to this challenge lies in the extension of the system’s applicability to video data, permitting a more comprehensive consideration of situational context.

In the case of Table 9c, our system accurately interpreted the presence of children lighting a fire and accordingly assessed the danger score as ‘7’. However, our system failed to detect the child lying on the railing to the right, which should have been identified. Table 9c exemplifies the limitations associated with situations where multiple scenarios occur simultaneously within a single scene. This table represents a scenario where distinct behaviors unfold concurrently, posing a challenge for detection. Describing such an image with a single caption is challenging. The solution to this challenge lies in integrating multi-object detection and dense captioning techniques. This integration allows for the generation of captions and sentences for multiple concurrently detected scenes, rather than attempting to fit multiple object and action descriptions into a single sentence.

## 5. Conclusions and Future Work

As the field of artificial intelligence advances and hardware capabilities improve, surveillance systems have evolved to handle a broad range of tasks. Our proposed surveillance system introduces a novel approach that empowers the system to autonomously interpret a wide spectrum of information, facilitating comprehensive situation analysis. This system leverages large multimodal models to generate descriptive captions for hazardous situations and employs semantic analysis to assess the associated risk levels effectively.

To create these captions, we incorporate object information, behavior details, and space context to monitor various situations, leveraging this information to measure risk. Our self-constructed dataset was designed to categorize risk levels based on factors such as the age group of individuals, types of actions, and the nature of locations. Through a series of experiments using these datasets, we demonstrate that they provide comprehensive information for risk assessment and exhibit exceptional performance in this regard. Compared to models pre-trained on existing datasets, our generated captions comprehensively encompass the requisite object attributes, behavior, and spatial context essential for the surveillance system. Furthermore, they exhibit adaptability to novel sentence structures, ensuring versatility across diverse contexts. The robustness of the dataset has also been evidenced by testing with images captured under various conditions, showing its adaptability to both indoor and outdoor environments. Consequently, monitoring personnel can make more accurate and quicker decisions by receiving combined information of the video, interpreted captions, and risk level assessment. Expanding our system to create caption data for additional situations can further enhance surveillance system performance, potentially culminating in a universally applicable system.

As part of our future research agenda, we plan to explore a system that combines multi-object detection and dense captioning technology to generate captions and seamlessly integrate sentences for multiple concurrently detected scenes. Furthermore, recognizing the constraints associated with detecting abnormal situations at the single-frame level, we aim to investigate the expansion of existing systems by incorporating video captioning technology that accounts for the context preceding and following an incident, thus enabling a more comprehensive and nuanced analysis.

## Figures and Tables

**Table 1:** Risk level and danger score classification criteria.

Risk Level	Danger Score	Classification Criteria	Object	Behavior	Space
Safe	1	In situations where objects within the image are located in a secure environment.	adult	walking, sitting, running, or riding	pathway, park, or indoors
	2		child		
Hazard	3	In situations where objects within the image are located in a hazard environment.	adult		railing, cliff, road, or a construction site
	4		child		
Danger	5	In situations where objects in the image engage in perilous activities.	adult, vehicle, or fire	collapse, fight, a fire breaks out, or traffic accident	pathway, park, or indoors
	6	In situations where objects within the image engage in perilous activities while located in a hazardous environment.			railing, cliff, road, or a construction site
	7	In situations where the subjects within the image are minors and are involved in perilous activities while situated in a hazardous environment.	child, vehicle, or fire		

**Table 2:** The quantity of datasets by each risk level and danger score.

Risk Level	Safe		Hazard		Danger			
Danger Score	1	2	3	4	5	6	7	
Number of Images	343	215	291	259	752	613	268	
Total	558		550		1633			2741

**Table 3:** The sentence structure format employed in the captions of our dataset.

Image	Caption	Risk Level (Danger Score)
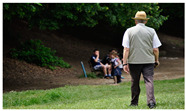	A man wearing a hat is walking in the park.	Safe (1)
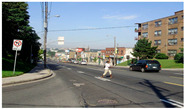	A man in a white shirt and cap is walking on the road.	Hazard (3)
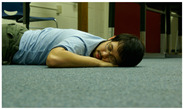	A man wearing a blue shirt is fainting in the room.	Danger (5)
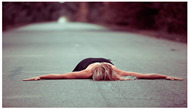	A woman wearing a black dress is lying down on the road.	Danger (6)

**Table 4:** The results of the BLEU scores for our dataset.

BLEU-1	BLEU-2	BLEU-3	BLEU-4
0.7062	0.5827	0.4873	0.4096

**Table 5:** The comparison results of caption generation between our system and other models.

	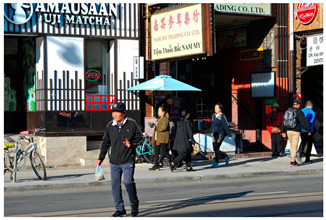 (a)	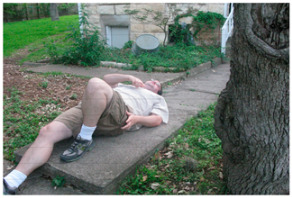 (b)
CPTR [15]	A man walking down a street with a skateboard.	A man lying on a park bench with his legs crossed.
OFA [16]	A man is crossing the street in a city.	A man lying on the ground next to a tree.
BLIP-2 [11] (Base)	A man walking down the street with an umbrella.	A man lying on the ground in front of a house.
Ours	A man in black pants is walking on the road.	A man wearing a tan T-shirt fell on the sidewalk.
	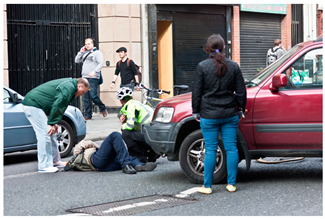 (c)	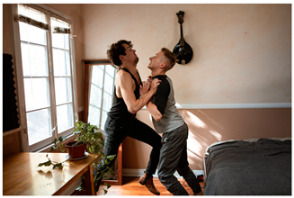 (d)
CPTR [15]	A man is standing next to a parked car.	A man and woman are standing in a room.
OFA [16]	A group of people standing around a man on the street.	Two men are dancing in a room.
BLIP-2 [11] (Base)	A man lying on the ground next to a red car.	Two young men are fighting in a bedroom.
Ours	A man wearing a jacket is lying next to a red car on the road, and a man in black is standing.	A man wearing a black top is fighting with a man wearing a gray shirt in the room.

**Table 6:** The results of risk assessment for each individual case.

	Danger Score (Ground Truth)	Image	Generated Caption	Predicted Danger Score
(a)	1	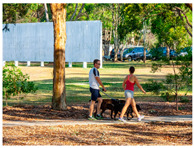	A man and woman are walking with a dog in the park.	1
(b)	2	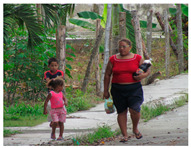	A woman in a red shirt is walking with two children on the street.	2
(c)	3	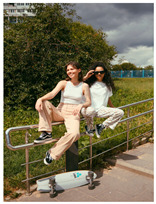	Two women are sitting on a railing.	3
(d)	4	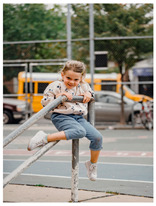	A girl wearing a white shirt is sitting on a railing.	4
(e)	5	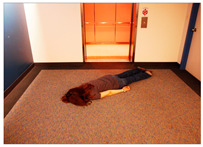	A woman wearing a gray t-shirt fell on the floor.	5
(f)	6	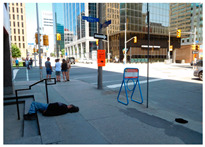	A woman wearing a blue shirt collapsed on the stairs.	6
(g)	7	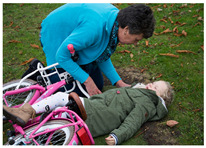	A girl in a green jacket fell next to a bicycle and a woman is helping her.	7

**Table 7:** Accuracy for three distinct risk levels on the test datasets.

	Output	Safety	Hazard	Danger	Number of Images	Accuracy
Ground Truth						
Safety		53	3	1	57	92.9%
Hazard		3	60	4	67	89.5%
Danger		1	7	169	177	94.3%
Total					301	93.0%

**Table 8:** Some experimental results that demonstrate the robustness of proposed surveillance system in unstable recording environments influenced by variations in lighting, shadows, and shooting resolution.

	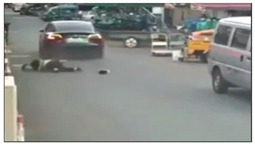 (a)	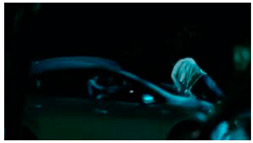 (b)	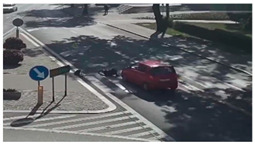 (c)
Influenced Factor	Low resolution	Low light condition	Overshadow
Caption	A man wearing a black top is lying on the road.	A man wearing a white top was hit by a car on the road.	A man falls down in a collision with a red car.
Danger Score	6	6	6

**Table 9:** Examples of distinct types of limitations and areas for improvement encountered by our system in the domains of image captioning and risk assessment.

	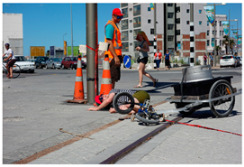 (a)	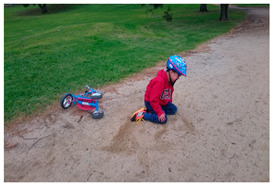 (b)	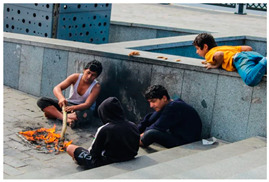 (c)
Caption	A man wearing a yellow safety vest is standing next to a bicycle on the road.	A boy wearing a helmet is sitting on the ground.	Three boys are burning a stick on the sidewalk.
Danger Score	3	2	7
Ground Truth	6	6	7
